# Transcriptomic Profiling Identifies a Distinct Molecular Signature in OSMF-Derived Oral Squamous Cell Carcinoma

**DOI:** 10.3390/life16030454

**Published:** 2026-03-10

**Authors:** Kavitha Prasad, Sowmya Samudrala Venkatesiah, Dominic Augustine, Ananya Anurag Anand, Prashanthi Karyala, Sukeerthi Dasharathy, Roopa S. Rao, Soma Chaki

**Affiliations:** 1 Department of Oral and Maxillofacial Surgery, Faculty of Dental Sciences, MS Ramaiah University of Applied Sciences, MSR Nagar, Bengaluru 560054, Karnataka, India; kavithaprasad.os.ds@msruas.ac.in; 2 Department of Oral & Maxillofacial Pathology and Oral Microbiology, Faculty of Dental Sciences, MS Ramaiah University of Applied Sciences, MSR Nagar, Bengaluru 560054, Karnataka, India; dominicaugustine.op.ds@msruas.ac.in (D.A.); roopa.op.ds@msruas.ac.in (R.S.R.); 3 Department of Applied Sciences, Indian Institute of Information Technology, Allahabad, Prayagraj 211012, Uttar Pradesh, India; ananyaanurag12@gmail.com; 4 Department of Biotechnology, MS Ramaiah University of Applied Sciences, Bengaluru 560054, Karnataka, India; prashanthi.bt.ls@msruas.ac.in (P.K.); sukeerthi.d96@gmail.com (S.D.); dean.ls@msruas.ac.in (S.C.)

**Keywords:** genomic landscape, mouth neoplasm, oral submucous fibrosis, transcriptome analysis

## Abstract

**Background:** Oral Submucous Fibrosis (OSMF) is a significant global oral health problem, particularly prevalent in India, with a high risk of progression to Oral Squamous Cell Carcinoma (OSCC). This study investigates the molecular mechanisms involved in the transformation of OSMF to OSCC using transcriptomic profiling. **Methods:** High-throughput RNA sequencing was performed on fresh de novo OSCC samples (*n* = 8) and OSMF derived OSCC using Illumina-compatible NEXTflex Rapid Directional RNA Sequencing. Normalization and differential gene expression analysis were conducted, and genes exhibiting an absolute log2 fold change of ≥2 with a co-variate-adjusted *p*-value ≤ 0.05 were identified as significant. **Results:** Upregulated genes were associated with cytokine and immune responses (ABRA, TTTY14, EIF1AY), cellular proliferation and apoptosis (LINC00314, RPS4Y1, SERPINA5, TRIM63, FABP7), and energy metabolism, indicating metabolic adaptations during malignant progression. Pathway analysis showed increased expression of TNNT1, TNNI1, MYL4, and ACTN3, implicating muscle development and embryonic pathways in OSMF transformation. Conversely, genes related to epithelial differentiation and keratinization (FLG, FLG2, HRNR, TCHH, KRT73), immune regulation and tumor suppression (HLA-G, UNC5D), and metabolic signaling were downregulated, reflecting loss of tissue integrity and immune control. **Conclusions:** OSMF-derived OSCC exhibits a distinct transcriptomic landscape compared with de novo OSCC, characterized by altered epithelial differentiation, immune modulation, and activation of developmental pathways. The observed gene dysregulation findings establish that OSCC developing in the background of OSMF is molecularly distinct from de novo OSCC, underscoring the biological impact of the pre-existing fibrotic milieu on tumor transcriptional architecture.

## 1. Introduction

Oral Submucous Fibrosis (OSMF) is a chronic, potentially malignant disorder characterized by inflammation and progressive fibrosis of the oral submucosa [1]. The global prevalence of OSMF is estimated at 4.96% (95% CI: 2.28–8.62%) [2]. The disease is predominantly reported in the Indian subcontinent and Southeast Asia, including India, Pakistan, and China. Prevalence varies widely depending on geographic region, sampling methods, and population size, ranging from 0.1% to 30% [3,4]. In India, reported prevalence ranges from 0.2–2.3% in males and 1.2–4.6% in females, while some studies report rates as high as 7.21% [5,6,7,8]. Higher prevalence has been observed in states such as Maharashtra, Andhra Pradesh, Karnataka, and Tamil Nadu. OSMF commonly affects individuals below 35 years, particularly men aged 20–40 years [9,10]. The primary etiological factor is areca nut or betel quid chewing, often combined with tobacco [10]. OSMF has a malignant transformation rate of approximately 6% (95% CI: 2–10), making early diagnosis and staging critical for management [11,12,13,14,15]. Current treatment modalities, unfortunately, have not demonstrated marked efficacy in the management of OSMF [16]. Hence, there exists a pressing imperative to establish prognostic and diagnostic criteria capable of accurately predicting OSMF transformation and its clinical consequences.

Recent molecular studies have revealed significant alterations in gene expression in OSMF. Diagnostic genes such as MYH6, TNNT3, MYL1, and TPM2 were upregulated in both saliva and tissue samples of early OSMF, suggesting novel non-invasive biomarkers [17]. Bioinformatic network analyses have identified hub genes associated with OSMF and progression to Oral Squamous Cell Carcinoma, implicating pathways involved in cell proliferation and muscle contraction [18]. Dysregulated microRNAs (e.g., miR-31, miR-21, miR-29b) and increased expression of markers like IGF-1R further support complex gene regulation in OSMF pathogenesis [19]. Additionally, genetic susceptibility factors such as GSTM1 and GSTT1 null polymorphisms have been associated with increased OSMF risk in South Asian populations [20]. Although transcriptomic alterations in conventional (de novo) OSCC have been extensively characterized, the molecular identity of OSCC arising in the background of Oral Submucous Fibrosis remains insufficiently defined. Existing studies have largely examined OSMF and OSCC independently or compared malignant tissue with normal mucosa, without directly interrogating whether OSMF-derived OSCC is transcriptionally distinct from de novo OSCC. Consequently, it remains unresolved whether malignant transformation in a chronically fibrotic microenvironment results in unique gene expression reprogramming or simply reflects the canonical molecular landscape of OSCC. This lack of direct comparative transcriptomic evidence represents a critical gap in understanding fibrosis-associated oral carcinogenesis [18,21].

Despite the antecedent endeavors in transcriptomic profiling among patients with head and neck cancer and oral squamous cell carcinoma (OSCC) [22], transcriptome analysis stands out for its role in pinpointing novel biomarkers crucial for early detection, prognostication of malignant transformation, and monitoring the disease’s responsiveness to medical interventions. The utilization of these studies offers manifold advantages, encompassing high-throughput and high-resolution data analysis, delineation of molecular signatures, insights into disease progression, and the potential for personalized medicine.

Therefore, the present study evaluates direct comparative transcriptomic analysis between OSMF-derived OSCC and de novo OSCC using high-throughput RNA sequencing. It also identifies differentially expressed genes and enriched molecular pathways that distinguish OSMF-associated OSCC from conventional OSCC. The study also determines whether the pre-existing fibrotic microenvironment is associated with context-specific transcriptional reprogramming, thereby supporting the concept that OSMF-derived OSCC represents a biologically distinct molecular subtype.

## 2. Materials and Methods

### 2.1. Sample Collection

The samples analyzed in this study included 5 histopathologically confirmed cases of OSMF-derived OSCC (Group 1) and 8 cases of frank de novo OSCC cases (Group 2). Among the 5 OSMF-derived OSCC cases, 3 were females and 2 were males. The 8 cases of frank de novo OSCC comprised 5 females and 3 males. The study group samples were derived from chronic areca nut and tobacco chewers diagnosed with OSMF reported to Ramaiah University of Applied Sciences, and M.S. Ramaiah Medical Teaching Hospital in Bangalore. Informed consent was obtained from all subjects involved in the study. Ethics clearance was obtained from the Institutional Ethics Committee. The collected fresh tissue specimens were preserved in RNA later and stored at −80 °C until the commencement of experimental procedures. Only histopathologically confirmed OSMF-derived OSCC and de novo OSCC were included in the study for transcriptomic analysis.

### 2.2. RNA Extraction

Total RNA extraction from the chosen samples was conducted employing an Isol + Qiagen RNeasy Mini Kit (QIAGEN, Hilden, North Rhine-Westphalia, Germany) in accordance with the manufacturer’s protocol (Invitrogen). Assessment of RNA concentration and purity was carried out using both a Nanodrop Spectrophotometer (Thermo Fisher Scientific, Waltham, MA, USA) and Qubit Fluorometer (Thermo Fisher Scientific, Waltham, MA, USA). To evaluate the RNA integrity (RIN) of the samples, an Agilent Bioanalyzer chip (Agilent Technologies, Santa Clara, CA, USA) was utilized, with a set criterion of RIN ≥ 7 for sample inclusion in the study. This stringent criterion ensured the selection of high-quality RNA samples for subsequent analyses. Data obtained represent bulk tissue transcriptomics.

### 2.3. Library Preparation

RNA sequencing libraries were meticulously prepared utilizing an Illumina-compatible NEXTflex Rapid Directional RNA-Seq Kit (BIOO Scientific, Austin, TX, USA). In a concise overview, approximately 1.5 μg of Qubit-quantified total RNA underwent PolyA enrichment. The resulting enriched mRNA underwent a 10 min fragmentation at 95 °C in the presence of divalent cations. Subsequent steps involved priming and reverse transcription using First Strand Synthesis Mix followed by the synthesis and end repair of the second-strand cDNA using the Second Strand Synthesis Mix. The maintenance of directionality was ensured by the incorporation of dUTP at this stage. The resultant double-stranded cDNA underwent purification using HighPrep PCR magnetic beads (Magbio Genomics Inc., Gaithersburg, MD, USA). Following purification, the cDNA was adenylated and ligated to Illumina multiplex barcode adapters in accordance with the NEXTflex Rapid Directional RNA-Seq Kit protocol. This methodical approach ensured the preparation of high-quality RNA sequencing libraries for downstream analyses.

### 2.4. The Illumina Universal Adapter

5′-AATGATACGGCGACCACCGAGATCTACACTCTTTCCCTACACGACGCTCTTCCGATCT-3′ and index adapter 5′-GATCGGAAGAGCACACGTCTGAACTCCAGTCAC*INDEX + ATCTCGTATGCCGTCTTCTGCTTG-3′ were employed in the preparation of sequencing libraries to identify sample-specific sequencing data. Following adapter ligation, the cDNA underwent purification using HighPrep beads (Magbio Genomics Inc., Gaithersburg, MD, USA) and subsequently underwent 10 cycles of polymerase chain reaction (PCR). The PCR conditions involved incubation at 37 °C for 30 min, denaturation at 98 °C for 2 min, cycling at 98 °C for 30 s, 65 °C for 30 s, and 72 °C for 60 s, followed by a final extension at 72 °C for 4 min. The resulting PCR product, constituting the sequencing library, underwent purification using HighPrep beads, and its quality was assessed through a library quality control check. Quantification of the Illumina-compatible sequencing library was initially performed using a Qubit fluorometer (Thermo Fisher Scientific, Waltham, MA, USA), while its fragment size distribution was further analyzed on an Agilent TapeStation (Agilent Technologies, Santa Clara, CA, USA) [22].

### 2.5. Bioinformatics Analysis of RNA Seq Data

#### 2.5.1. Upstream RNASeq Analysis

Total RNA was used for mRNA sequencing, and RNA-seq libraries were prepared following standard Illumina protocols (poly-A mRNA enrichment) and sequenced on the Illumina NovaSeq platform to generate paired-end FASTQ files.

Raw FASTQ files were imported into the R environment using the ShortRead package (Bioconductor) for initial quality assessment, including evaluation of per-base sequence quality, GC content, and read length distribution [23,24]. Adapter contamination and low-quality reads were filtered using the Rfastp package with default parameters, retaining only high-quality reads for downstream analysis.

Post-filtered reads were aligned to the UCSC hg19 (GRCh37) reference genome (Homo sapiens) [25]. A SAF-formatted exon annotation file was used to guide accurate genomic feature mapping during alignment. Alignment quality was assessed based on mapping rate and read distribution across genomic features [24].

Gene-level expression quantification was performed by counting aligned reads [24]. Read counts were generated using the Genomic Alignments package, while exon-level counts were obtained using the Genomic Features package. Feature-based read summarization was performed to obtain raw gene counts for each sample.

#### 2.5.2. Downstream RNASeq Analysis

Differential gene expression analysis was conducted using the DESeq2 package in the R Bioconductor framework [26,27]. Raw read counts were normalized using DESeq2’s median-of-ratios method to account for sequencing depth and library size variations. Pairwise comparisons were performed between experimental groups. Genes with an absolute log2 fold change (|log2FC|) ≥ 2 and an adjusted *p*-value (Benjamini–Hochberg’s correction) ≤ 0.05 were considered significantly differentially expressed genes (DEGs) [16].

The present study represents an exploratory transcriptomic analysis. While the sample size is limited due to the rarity of OSMF-derived OSCC samples, stringent statistical thresholds (log2 fold change of ≥2) and multiple testing correction (*p*-adjusted) were applied to ensure robustness of differential expression results [25,26,27]. The findings are intended to generate biologically relevant hypotheses that warrant validation in larger independent cohorts.

Gene annotation and conversion of Ensembl gene identifiers to gene symbols were carried out using the org.Hs.eg.db annotation database [16,28,29].

### 2.6. Gene Ontology and Pathway Analysis

To investigate the biological significance of the identified DEGs, Gene Ontology (GO) and pathway enrichment analyses (using KEGG) were performed [28,29]. The list of significantly upregulated and downregulated genes was used as input for functional enrichment analysis.

Statistically enriched biological processes, molecular functions, and cellular components (GO terms), as well as pathway annotations including KEGG pathways, canonical pathways, and hallmark gene sets, were identified [28,29]. Enrichment analysis was conducted using a hypergeometric test, and both enrichment factors and cumulative *p*-values were calculated. Multiple testing correction was applied, and terms with adjusted *p*-values ≤0.05 were considered significantly enriched.

Further GO enrichment analysis was applied to elucidate functional networks and biological processes associated with DEGs, enabling interpretation of the molecular mechanisms underlying the observed transcriptional changes. This approach provided a comprehensive functional overview of DEG-associated biological pathways and processes [22]. However, it is important to remember that overlapping gene annotation within hierarchical annotation systems leads to recurrent enrichment of related GO and pathway terms and indicates coordinated transcriptional signatures rather than independent biological function [30,31].

## 3. Results

### 3.1. Transcriptome Analysis Strategy for Identifying the Progression of OSMF to OSCC

To compare transcriptomic alterations associated with the progression of Oral Submucous Fibrosis (OSMF) to Oral Squamous Cell Carcinoma (OSCC) with those observed in de novo OSCC, RNA sequencing analysis was performed on 13 human oral tissue samples derived from Groups 1 and 2. The dataset comprised an average of 37,306,577 and 35,885,962 raw sequencing reads for Groups 1 and 2, respectively. Following quality filtering, an average of 45,043,233 high-quality processed reads per group were retained and aligned to the UCSC hg19 (GRCh37) reference genome of Homo sapiens. Transcriptome sequencing was carried out using the Illumina HiSeq platformIllumina HiSeq platform 2500 (Illumina Inc., San Diego, CA, USA). Differential expression analysis identified a total of 1146 differentially expressed genes (DEGs), including 630 upregulated and 516 downregulated genes.

### 3.2. Differentially Expressed Genes in OSMF-Derived OSCC vs. De novo OSCC

The differentially expressed genes were identified among Groups 1 and 2. Among the OSMF-derived OSCC group vs. de novo OSCC, 1146 DEGs were identified, of which 630 were upregulated and 516 downregulated. The top upregulated and downregulated genes are documented in Table 1. The deregulatedgenes involved in biological and cellular processes are demonstrated in Table 2, Table 3 and Table 4. The observed deregulation between Group 1 (OSMF-derived OSCC) and Group 2 (OSCC samples) highlights significant changes in gene expression. Several genes exhibit notable upregulation in OSCC samples compared to those transformed to OSCC from OSMF. Noteworthy upregulated genes include LINC00314, ABRA, TTTY14, CACNG7, RPS4Y1, SERPINA5, TRIM63, EIF1AY, etc. Cytokine- and immune-response-related genes (ABRA, TTTY14, EIF1AY, LRP1B, KRT76, TNNI1, TYRP1, SLC25A4, ZFY, HRCT1, MT1M), cellular-proliferation- and apoptosis-related genes (RPS4Y1, SERPINA5, TRIM63, RPL3L, FABP7, COX7A1, SBK3), ECM-turnover-related genes (MMP-3, MMP-9), and transcription-factor- and signaling-pathway-related genes (MYL2 and CSF3) were majorly upregulated. Genes involved in cellular differentiation and keratinization (KRT73, FLG, FLG2, TCCH), immune regulation and tumor suppression (HLA-G, GPR22, UNC5D) and metabolic and signaling pathways (SLC6A10P, GUCY1B2 and CXADRP3) were majorly downregulated. Differential expression of Y-linked genes can arise due to sex distribution differences rather than disease-specific biology.

### 3.3. Gene Ontology and Pathway Enrichment Analysis

**Figure 1:**
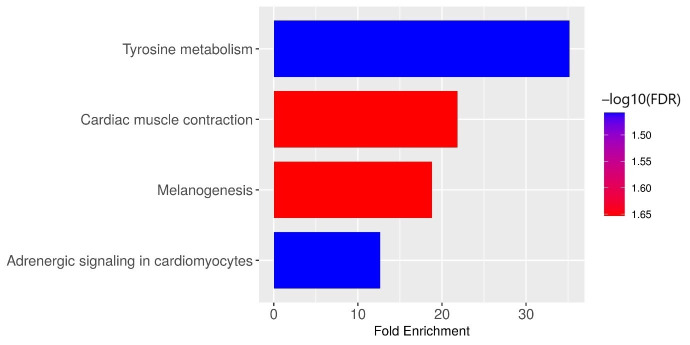
KEGG pathway enrichment of upregulated genes in OSMF-derived OSCC vs. de novo OSCC. Kyoto Encyclopedia of Genes and Genomes (KEGG) pathway enrichment analysis was conducted on significantly differentially expressed genes (|log_2_ fold change| ≥ 2 and adjusted *p*-value ≤ 0.05) to identify overrepresented biological pathways. Enrichment analysis was performed using a hypergeometric test followed by the Benjamini–Hochberg correction for multiple comparisons. Pathways with adjusted *p*-values ≤ 0.05 were considered significantly enriched.

The upregulated genes in OSMF derived OSCC group subjected to KEGG pathway analysis identified tyrosine metabolism, cardiac muscle contraction, melanogenesis and adrenergic signaling in cardiomyocytes as the major deregulated pathways (Figure 1).

This figure illustrates the top KEGG pathways significantly enriched among upregulated genes in OSMF-derived OSCC compared to de novo OSCC. Notable pathways include tyrosine metabolism, cardiac muscle contraction, melanogenesis, and adrenergic signaling, indicating alterations in energy regulation, pigmentation, and cellular adaptation during malignant transformation. Genes such as DCT, CACNG7, MYL4, CALML6, and TYRP1 were prominently involved in these pathways. Genes including DCT, CACNG7, MYL4, CALML6, and TYRP1 were significantly enriched.

**Figure 2:**
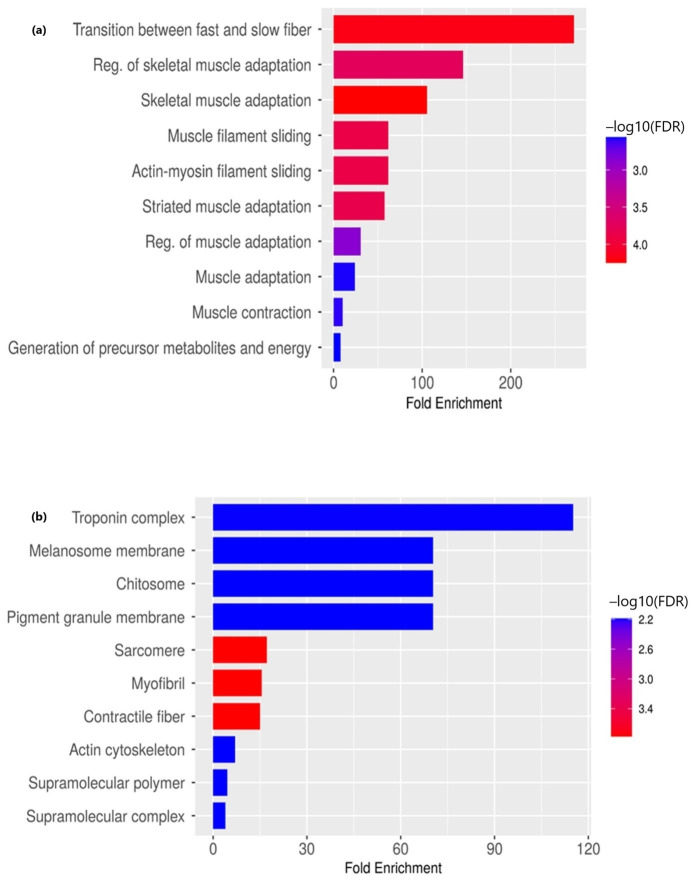
Gene Ontology enrichment of upregulated genes in OSMF-derived OSCC vs. de novo OSCC. (**a**) Biological processes and (**b**) cellular components. Gene Ontology (GO) enrichment analysis was performed for significantly differentially expressed genes (|log_2_ fold change| ≥ 2 and adjusted *p*-value ≤ 0.05). Enrichment analysis was conducted using a hypergeometric test, and multiple testing correction was applied using the Benjamini–Hochberg method. GO terms with adjusted *p*-values ≤ 0.05 were considered significantly enriched.

The pathways related to the transition between fast and slow fiber, muscle adaptation, contraction and filament sliding were the major hits for biological processes associated with upregulated genes. Upregulated genes in OSMF-derived OSCC were enriched in processes associated with skeletal muscle function and adaptation, including regulation of fiber type switching, filament sliding, and muscle contraction. This suggests potential tissue remodeling or cytoskeletal reorganization during disease progression. The enrichment reflects tumor–muscle interface microenvironmental contribution rather than intrinsic epithelial expression. There was a significant expression of TNNT1, TNNI1, ACTN3, MYL4 and TRIM63 genes in the above-mentioned process (Figure 2a; Table 2). The cellular component analysis revealed the same set of upregulated genes associated with fundamental muscle structures like troponin complex, sarcomere, myofibril, and contractile fiber helpful in muscle contraction and integrity. There was a significant expression of genes involved in pigmentation-related processes like melanin biosynthesis (DCT, TYRP1), underscoring a potential connection between pigmentation process and muscle biology (Figure 2b). Our study did not identify any significant hits for the molecular function of upregulated genes.

**Figure 3:**
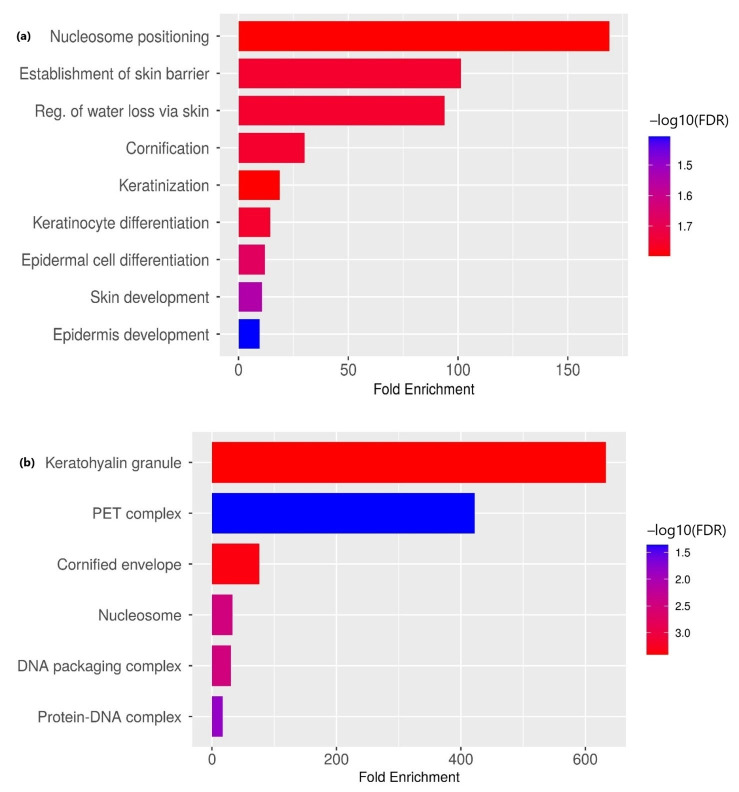
Gene Ontology enrichment of downregulated genes in OSMF-derived OSCC vs. de novo OSCC. (**a**) Biological processes and (**b**) cellular components. Gene Ontology (GO) enrichment analysis was performed for significantly differentially expressed genes (|log_2_ fold change| ≥ 2 and adjusted *p*-value ≤ 0.05). Enrichment analysis was conducted using a hypergeometric test, and multiple testing correction was applied using the Benjamini–Hochberg method. GO terms with adjusted *p*-values ≤ 0.05 were considered significantly enriched.

The results indicate significant downregulation of genes implicated in biological processes, including skin development, barrier function and epidermal cell differentiation (Figure 3a; Table 2). This suggests weakening of the mucosal barrier and structural changes that may facilitate malignant invasion. Key downregulated genes include FLG, HRNR, KRT73, and TCHH (Figure 3a; Table 3). This downregulation could potentially also disrupt nucleosome positioning, compromising chromatin organization and gene expression regulation. The downregulated genes, such as FLG, HRNR, H1-1, H1-5 and H2AC12, were significantly enriched with keratohyalin granule, PET complex, cornified envelope, nucleosome, DNA packaging complex, and protein–DNA complex cellular components (Figure 3b, Table 3). This reflects potential disruption of chromatin organization and epithelial structure during malignant progression. Table 4 depicts the clinical and pathological characteristics of all the samples. The staging was ascertained via positron emission tomography–computed tomography (PET-CT).

## 4. Discussion

Globally, oral cancer ranks as the sixth most prevalent malignancy, with India bearing a significant burden, contributing to nearly one-third of total cases and being the second-highest country in terms of incidence [16]. Annually, India reports approximately 77,000 new cases and 52,000 deaths due to oral cancer, constituting roughly one-fourth of the global caseload. This escalating prevalence underscores a critical public health challenge, particularly in India, where oral cancer is among the most frequently diagnosed cancers [32]. Notably, compared to Western nations, India experiences a disproportionately higher prevalence of advanced-stage oral cancer cases, with around 70% of diagnoses occurring in later stages [33]. Consequently, late-stage detection severely diminishes the prospects of successful treatment, resulting in a dismal five-year survival rate of approximately 20% [34]. Various risk factors contribute to the incidence of oral cancer, including tobacco consumption in its various forms such as smokeless tobacco and betel quid chewing, along with excessive alcohol intake and poor oral hygiene practices. These factors, coupled with a lack of awareness, exposure to harsh environmental conditions, and behavioral aspects, exhibit significant global variability in oral cancer rates [35]. Additionally, periodontal diseases represent a notable risk factor for oral malignancies, particularly prevalent among the Indian population where the habit of chewing paan is widespread. This habit entails prolonged exposure of the oral mucosa to irritants, leading to epithelial abrasion and often resulting in OSMF, thereby increasing the susceptibility to oral cancer [33].

OSMF is characterized by epithelial atrophy, potentially facilitating the penetration of carcinogens [36]. Arecoline, a component of betel nut, acts as a desiccating agent, inducing cellular shrinkage and potentially enhancing the permeability of the epithelium to carcinogens, allowing their access to the basal layer. This basal layer, being the site of cell division, is susceptible to neoplastic transformation. The increased permeability of the epithelium to carcinogens represents a significant mechanism in arecoline-associated carcinogenesis [37]. Research indicates that approximately 25% of biopsied OSMF cases exhibit dysplasia, highlighting the oncogenic potential of this condition [36]. Epithelial dysplasia is documented in varying proportions, ranging from 7% to 43%, among individuals with OSMF across different research studies [37]. However, the transformation rate to malignancy exhibits variability, ranging from 3% to 19% [37,38]. Notably, recent findings from a study conducted in India revealed a concurrent occurrence of OSCC with OSMF at a rate of 25.77%. This suggests that the malignant potential of OSMF is frequently underestimated [39]. Given that areca nut consumption triggers a distinct molecular pathway in oral carcinogenesis, it is plausible that individuals presenting with both OSMF and OSCC exhibit diverse morphological, histological, and biological characteristics [40]. Presently, there is a proposition that OSCC emerging within the context of OSMF represents a clinicopathologically distinct entity. These differences are postulated to stem from varying mechanisms involved in areca nut carcinogenesis [41,42]. Given the ongoing debate within the literature concerning OSCC development in the backdrop of OSMF, our current study employs gene expression profiling to identify upregulated and downregulated genes. This approach aims to elucidate distinct molecular pathways implicated in OSCC compared to OSMF.

The current investigation delves into the intricate molecular mechanisms underlying the transition from Oral Submucous Fibrosis (OSMF) to Oral Squamous Cell Carcinoma (OSCC), revealing a multifaceted interplay of genetic processes and pathways pivotal in the progression of oral cancer. The observed upregulation of specific genes associated with muscle development (e.g., ABRA, TRIM63, ACTN3, MYL4, TNNT1, TNNI1, MYL6B), embryonic pathways (e.g., PITX3, NKX2-3), and energy metabolism (e.g., EIF1AY, FABP7, COX7A1, ENO3, GYS2, HRCT1, FXYD1) signifies heightened cellular proliferation, tissue remodeling, and metabolic reprogramming—hallmark features indicative of malignant transformation. The observed upregulation of genes associated with epithelial characteristics (e.g., KRT76) and melanin synthesis (e.g., DCT, TYRP1) signifies potential alterations in cellular behavior and pigmentation dynamics. Concurrently, the heightened expression of genes involved in cellular signaling and interaction (e.g., SERPINA5, LRP1B, TNNT1, HS3ST2, SLC25A4) suggests enhanced intercellular communication, exerting a significant influence on tumor growth. Moreover, dysregulated transcriptional regulation and upregulation of genes related to mitochondrial function contribute to the modification of cellular phenotypes. Furthermore, specific genes are implicated in various critical processes, including calcium signaling, pH regulation, lipid metabolism, membrane dynamics, cyclic AMP signaling, DNA damage response, and histone modification, underscoring the diverse molecular mechanisms underlying oral cancer progression.

The identification of downregulated genes during the transition from OSMF to OSCC provides valuable insights into the molecular changes associated with disease progression. Genes involved in epigenetic regulation and transcriptional control (e.g., MKRN9P, BRD7P3, TSIX, H2BP1) may influence gene expression patterns and chromatin dynamics. Moreover, the downregulation of immune-related genes (e.g., HLA-G, GPR22, SCARNA10) indicates a compromised immune response, potentially contributing to immune evasion mechanisms. Similarly, the reduced expression of genes involved in cell cycle regulation and tumor suppression (e.g., BAGE3, TFAMP1, FLG, CCNYL6) suggests a potential loss of control over cellular proliferation. The decreased expression of metastasis suppression genes (e.g., UNC5D, HRNR, SCN2A) enhances the likelihood of metastatic dissemination. Collectively, these findings shed light on the molecular alterations underlying the progression from OSMF to OSCC. The downregulation of genes associated with cytoskeletal organization and differentiation (e.g., FLG2, KRT25, TCHH) has implications for cell structure and differentiation processes. Additionally, the decreased expression of genes involved in extracellular matrix (ECM) interaction and signaling (e.g., ANKRD34B, SLCO1A2, STRA8) affects cellular adhesion, migration, and invasion dynamics. The observed downregulation of olfactory receptor signaling genes (e.g., TAS2R43, LOC442028) indicates potential alterations in chemosensory functions. Moreover, the downregulation of RSPO1, a gene linked to Wnt signaling, may influence both cell adhesion and signaling pathways associated with cancer development. These findings collectively underscore the multifaceted molecular alterations contributing to the progression of oral cancer.

One of the upregulated genes, ABRA (Actin-Binding Rho Activating), plays a crucial role in regulating actin dynamics, which in turn affects various cellular processes including migration, invasion, and metastasis [43]. Rho GTPases, such as Rho, Rac, and Cdc42, are pivotal for the formation of actin-rich structures like lamellipodia, filopodia, and stress fibers, which facilitate the mobility and invasion of cancer cells [44]. Additionally, actin-binding proteins (ABPs) like Mena/VASP, SATB1, and WASP, among others, are implicated in the early stages of carcinogenesis by modulating the expression of oncogenes and regulating actin polymerization. These molecular mechanisms underscore the intricate role of ABRA and associated actin-binding proteins in cancer progression [44]. These proteins collaborate with Rho GTPases to regulate the organization of the actin cytoskeleton, a process crucial for cancer progression. In particular, Rho GTPases such as RhoA activate formin mDia, which initiates the nucleation of unbranched actin filaments. This activity promotes the formation of actin stress fibers and focal adhesions. The interplay between Rho GTPases and their downstream effectors dictates the level of actin polymerization, thereby influencing various cellular behaviors including migration, invasion, and metastasis [43,44].

In a study conducted by Gong et al., six long non-coding RNAs (LncRNAs) were identified in laryngeal squamous cell carcinoma, namely LINC02154, LINC00528, SPRY4-AS1, TTTY14, LNCSRLR, and KLHL7-DT [38]. In our study, LINC00314 was significantly upregulated with a log2 fold change of 25.53458, which was approximately 3 times the other top upregulated genes. The significant upregulation of LINC00314 in our study suggests an active role of this lncRNA in cellular processes, potentially impacting the pathogenesis of OSMF. LINC00314, a long non-coding RNA, has been implicated in the regulation of the Wnt signaling pathway, which is a crucial mediator of cell proliferation, differentiation, and migration. A study by Shi et al. has identified LINC00314 as a long non-coding RNA (lncRNA) that plays a role in facilitating osteogenic differentiation. The study reported the involvement of LINC00314 in the stimulation of osteogenic differentiation of adipose-derived stem cells (ADSCs) through the hsa-miR-129-5p/GRM5 axis via the Wnt signaling pathway, and it indirectly influences the expression of genes involved in cancer progression [1]. A study by Yu et al. reported similar findings and reported that LINC00314 acts as a sponge of miR-129-5p that has been demonstrated to act as an oncogene or tumor suppressor in various cancers like retinoblastoma, non-small-cell lung cancer, and hepatocellular carcinoma [3].

TTTY14, in particular, has been observed to be downregulated in OSCC. Notably, patients exhibiting high levels of TTTY14 expression demonstrate a prolonged survival time. Cox’s multivariate analysis has further demonstrated that low expression of TTTY14 serves as an independent prognostic risk factor for OSCC patients [45]. Interestingly, our study revealed an upregulation of TTTY14, which contrasts with its typically downregulated expression in gastric cancer. Notably, low expression of TTTY14 is associated with an unfavorable prognosis in gastric cancer patients as well. TTTY14 is involved in constructing a prognostic model for gastric cancer patients [46]. Previous studies have reported the expression of TTTY14 in patients infected with human papillomavirus (HPV), and there is emerging evidence indicating HPV infection as a significant risk factor in OSMF [47]. Moreover, analysis conducted using data from the Human Protein Atlas has demonstrated a notable correlation between elevated expression levels of CACNG7 (Calcium Voltage-Gated Channel Auxiliary Subunit Gamma 7) and patient survival across diverse cancer types, including gastric cancer [48]. Notably, our investigation identified an upregulation of CACNG7, suggesting its potential utility as a prognostic indicator in the context of gastric cancer. However, its role in oral cancer does not have any substantial evidence.

In our study, the expression of Makorin Ring Finger Protein 9 (MKRN9) was observed to be downregulated. Conversely, Zhang et al. reported elevated expression of MKRN3 in squamous cell carcinoma of the head and neck. Presently, MKRN3 is recognized as a novel imprinted gene implicated in the progression of osteosarcoma and non-small-cell lung cancer. However, the precise role of MKRN3 in OSMF remains to be elucidated, particularly in terms of its association with clinical outcomes [49]. Salama et al. conducted a study identifying two long non-coding RNAs (lncRNAs), XIST and TSIX, known to play pivotal roles in X-chromosome inactivation, as potential biomarkers in breast cancer [50]. Interestingly, our study revealed a downregulation of these genes. We note that certain differentially expressed genes identified in this study are sex-chromosome-linked, including Y-chromosome-encoded genes such as EIF1AY and RPS4Y1, as well as X-chromosome-associated genes including XIST and TSIX. These genes were not excluded from the analysis, as their differential expression may reflect underlying biological and sex-specific regulatory differences between the study groups. However, given the limited sample size and potential imbalance in sex distribution, the expression patterns of sex-linked genes were interpreted with caution, and their contribution to disease progression warrants further validation in larger, sex-matched cohorts.

Additionally, the RSPO (R-spondin) family, consisting of four proteins (RSPO1-4), serves as crucial secreted regulators of the Wnt/β-catenin and Wnt/PCP signaling pathways. Alterations in RSPO, primarily through gene fusions or upregulation, have been reported in various cancer types, including tongue squamous cell carcinoma [51].

Melanin synthesis and the presence of melanocytes are known to confer a protective function against chemotoxic agents, particularly those present in tobacco products. Variations in melanocyte density and the distribution of melanin granules across different grades of oral epithelial dysplasia suggest a potential defense mechanism against harmful chemicals. Evaluating alterations in melanocyte density and melanin granules throughout the progression of oral epithelial dysplasia is crucial for comprehending their role in disease advancement [51]. In our study, we observed elevated expressions of dopachrome tautomerase (DCT) and tyrosinase-related protein 1 (TYRP1). Both enzymes play crucial roles in melanin biosynthesis. Specifically, dopachrome tautomerase (DCT) catalyzes the conversion of dopachrome to 5,6-dihydroxyindole-2-carboxylic acid (DHICA), a precursor of eumelanin [52]. On the other hand, TYRP1 competes with dopachrome for dopaquinone, which is the substrate of DCT. TYRP1 acts as a negative regulator of melanin production by inhibiting dopachrome conversion [53]. Understanding the upregulation of these two enzymes in oral cancer may provide insights into the potential role of melanin synthesis and melanocytes in disease progression.

The enhanced biological processes identified during the transition from OSMF to OSCC in our study provide valuable insights into the molecular adaptations occurring during disease progression. These processes encompass a range of activities essential for tissue remodeling and functional restoration.

The enrichment of muscle-development- and contractile-pathway-associated genes observed in OSMF-derived OSCC warrants cautious interpretation. While these pathways are traditionally linked to skeletal muscle differentiation, their activation in the present context is unlikely to represent true muscle tissue restoration within the oral epithelium. Instead, several biologically plausible explanations may account for this finding [54]. First, the chronic fibrotic microenvironment characteristic of OSMF is marked by abundant myofibroblasts, which express contractile proteins and cytoskeletal components that overlap with skeletal and striated muscle gene signatures. Thus, the upregulation of genes such as TNNT1, TNNI1, MYL4, and ACTN3 may reflect stromal activation and extracellular matrix remodeling rather than epithelial muscle differentiation per se [55].

Second, bulk RNA sequencing inherently captures signals from both tumor epithelial cells and surrounding stromal compartments, including fibroblasts and inflammatory cells. Therefore, stromal contribution or mesenchymal enrichment may partially explain the observed pathway activation [56]. Third, reactivation of developmental or contractile gene programs has been reported in several malignancies and may represent lineage plasticity or epithelial–mesenchymal reprogramming rather than functional muscle adaptation. Such transcriptional shifts may reflect broader cytoskeletal remodeling and microenvironment-driven alterations associated with tumor progression within fibrotic mucosa [57].

Accordingly, these findings should be interpreted as indicative of fibrosis-associated transcriptional reprogramming rather than evidence of organized muscle regeneration. Further studies using spatial transcriptomics or single-cell approaches would be necessary to delineate the cellular origin and functional significance of these gene signatures.

A substantial body of literature has characterized dynamic alterations in gene expression across the continuum from normal mucosa through potentially malignant disorders to Oral Squamous Cell Carcinoma (OSCC) [58]. These studies have identified dysregulation of key genes involved in cell cycle control, apoptosis, epithelial–mesenchymal transition, and extracellular matrix remodeling, thereby supporting the biological relevance of transcriptomic changes in oral carcinogenesis and providing a foundation for the present work [59].

For example, alterations in keratinocyte differentiation markers such as TP53, CDKN2A (p16), and CCND1 have been consistently reported in OSCC relative to normal mucosa. Incremental changes in expression of matrix metalloproteinases (MMP1, MMP9) and growth factor signaling components (EGFR, TGFB1) have also been documented with progression from dysplasia to carcinoma [60]. Large-scale expression profiling studies using microarrays or RNA sequencing have further elucidated gene signatures linked to malignant transformation and clinical outcomes in oral cancer [61]. These prior reports demonstrate that mRNA expression changes are integral to oral cancerogenesis and substantiate the rationale for focusing on transcriptomic alterations in our study.

**Figure 4:**
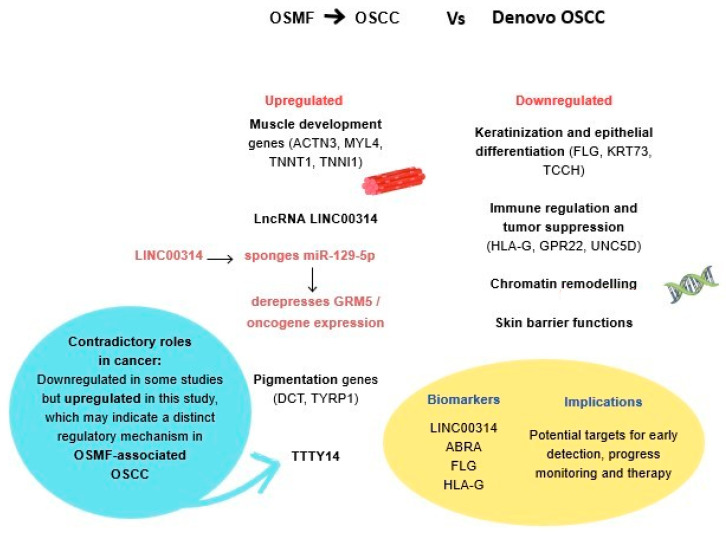
Integrated molecular map of OSMF-derived OSCC vs. de novo OSCC.

The downregulated processes encompass critical activities that are vital for maintaining tissue integrity and function. These include nucleosome positioning, establishment of the skin barrier, regulation of water loss via the skin, cornification, keratinization, keratinocyte differentiation, epidermal cell differentiation, skin development, and epidermis development. These processes are fundamental for both skin and mucosal health. In the context of OSMF, the downregulation of these processes may indicate disruptions in normal skin and mucosal functions. Nucleosome positioning, for instance, is crucial for gene regulation and chromatin organization, and its downregulation may lead to altered gene expression patterns [41]. The establishment of the skin barrier is essential for protecting against external insults and maintaining homeostasis. In the context of OSMF, downregulation of this barrier could compromise its function in affected tissues. Similarly, the regulation of water loss via the skin is crucial for maintaining hydration levels [42]. Dysregulation of this process may contribute to dryness and impaired healing in OSMF. Additionally, cornification, keratinization, and keratinocyte differentiation are fundamental processes involved in skin barrier formation and maintenance. The downregulation of these processes could lead to compromised barrier integrity in OSMF-affected tissues. Additionally, epidermal cell differentiation, skin development, and epidermis development are vital for tissue regeneration and maintenance. The downregulation of these processes may impede the repair mechanisms required to reverse the effects of OSMF. The collective decrease in these processes indicates a complex interaction of molecular changes contributing to the pathogenesis of OSMF. A molecular map of OSMF-derived OSCC vs. de novo OSCC is summarized in Figure 4.

Previous transcriptomic studies in OSCC have consistently demonstrated dysregulation of pathways related to epithelial differentiation, immune modulation, cell proliferation, extracellular matrix remodeling, and metabolic reprogramming. Similarly, molecular investigations in OSMF have highlighted fibrosis-associated signaling, including TGF-β activation, collagen deposition, inflammatory mediators, and oxidative stress pathways. However, most studies have examined OSMF and OSCC independently or compared diseased tissues with normal mucosa without directly evaluating whether OSCC arising in a fibrotic background differs from de novo OSCC at the transcriptomic level [62].

Our findings align with established OSCC signatures, particularly the suppression of epithelial differentiation markers and alterations in immune and metabolic pathways. At the same time, the enrichment of muscle-associated and developmental gene programs in OSMF-derived OSCC suggests additional context-specific transcriptional changes that may reflect stromal activation, myofibroblast contribution, or fibrosis-associated reprogramming. Such features are less prominently described in conventional OSCC transcriptomic datasets [63].

Thus, rather than diverging from the prior literature, our study extends it by introducing a direct comparative framework. The data support the concept that while OSMF-derived OSCC retains core oncogenic pathways characteristic of OSCC, the fibrotic microenvironment may imprint additional transcriptional features, contributing to a context-influenced molecular phenotype. Further validation using cell-type-resolved approaches will be necessary to delineate the precise cellular origins of these signatures [64].

Understanding the implications of these dysregulated processes in the pathogenesis of OSMF is pivotal for devising targeted interventions that aim to restore normal tissue functions and deter disease progression [65]. Further investigation into these molecular alterations holds promise for identifying novel therapeutic targets, addressing the intricate pathophysiology of OSMF, and potentially preventing its progression to OSCC.

This work was designed as an exploratory, discovery-based transcriptomic analysis to identify molecular signatures distinguishing OSMF-derived OSCC from de novo OSCC. The strengths highlighted include the comprehensive transcriptomic profiling and robust bioinformatic analyses that provide valuable insights into dysregulated genes and oncogenic pathways. A few limitations, such as a small cohort size, lack of external validation, sex imbalance in sampling and non- inclusion of comparisons of normal oral mucosa and OSMF cases, are a part of this study. They also include the reliance on mRNA-level analysis, which may not directly reflect protein expression or functional activity, the inability to capture post-transcriptional and translational regulatory mechanisms, the exclusion of other regulatory layers such as non-coding RNAs, and the potential influence of cell type heterogeneity inherent to bulk transcriptomic data. We emphasize that future studies should aim at validating and consolidating the functional roles of the identified genes and pathways, including in vitro functional assays such as gene knockdown or overexpression studies, cell proliferation, migration, and invasion assays, as well as pathway-specific inhibition or activation experiments. Studies in future with larger, balanced cohorts should explore sex-stratified transcriptomic patterns more comprehensively. Additionally, future studies incorporating in vivo models and larger independent cohorts are suggested to further substantiate the biological and clinical significance of these molecular alterations.

## 5. Conclusions

By employing transcriptomic analysis, we identified key genetic alterations and dysregulated pathways associated with OSMF-derived OSCC, highlighting potential molecular markers and pathways that warrant further investigation as candidate diagnostic or therapeutic targets. Although cross-sectional in design, the comparison between OSMF-derived OSCC and de novo OSCC captures molecular differences associated with malignant transformation in the context of OSMF at a population level. Among the differentially expressed genes, those involved in cytokine and immune responses, such as ABRA, TTTY14, and EIF1AY, and cellular-proliferation- and apoptosis-related genes, such as LINC00314, RPS4Y1, SERPINA5, TRIM63, FABP7, COX7A1, and SBK3, were significantly upregulated. Genes associated with cellular differentiation and keratinization, such as KRT73, FLG, FLG2, and TCCH, immune regulation and tumor suppression, such as HLA-G, GPR22, and UNC5D, and metabolic and signaling pathways (SLC6A10P, GUCY1B2, and CXADRP3) were predominantly found to be downregulated. In the pathway enrichment analysis, the upregulation of genes associated with muscle development, embryonic pathways, and energy metabolism such as TNNT1, TNNI1, MYL4, and ACTN3 underscores the intricate progression of cancer from OSMF. Conversely, the downregulation of genes involved in preserving tissue integrity and function such as FLG, HRNR, TCHH, and KRT73 explains molecular alterations that contribute to weakened epithelial barrier, promoting invasion and oral cancer development. Further validation of the identified biomarkers and pathways in larger patient cohorts is imperative to enhance their clinical relevance and applicability. Additionally, integrating multi-omics approaches, including genomics, proteomics, and metabolomics, could offer a comprehensive understanding of the molecular landscape and heterogeneity

## Figures and Tables

**Table 1:** Comparison of fold change in the upregulated and downregulated genes between OSMF-derived OSCC and de novo OSCC groups.

Upregulated Gene Name	log2 Fold Change	Downregulated Gene Name	log2 Fold Change
LINC00314	25.53458	MKRN9P	−3.94878
ABRA	8.156859	BRD7P3	−3.97669
TTTY14	7.113995	TSIX	−3.97867
CACNG7	6.837856	H2BP1	−3.99511
NA	6.51264	RSPO1	−4.08716
RPS4Y1	6.271099	SLC6A10P	−4.08729
SERPINA5	6.123807	SCARNA10	−4.09661
TRIM63	6.121181	KRT73	−4.23141
EIF1AY	6.095857	CXADRP3	−4.25731
DCT	5.936138	GUCY1B2	−4.2845
MIR4444-1	5.919456	MTRNR2L1	−4.30327
CALML6	5.868331	RFPS26P11	−4.36663
PITX3	5.765851	NA	−4.39118
NKX2-3	5.667237	HLA-G	−4.42497
CA4	5.644722	GPR22	−4.44261
FABP7	5.638956	BAGE3	−4.50707
COX7A1	5.60598	RPL31P11	−4.53903
SBK3	5.540832	ANKRD30BL	−4.55916
MYL4	5.527644	TFAMP1	−4.56978
LINC02067	5.523998	FLG	−4.59408

**Table 2:** Comparison of biological processes of deregulated genes between OSMF-derived OSCC and de novo OSCC groups.

Upregulated			Downregulated		
Pathway	Enrichment	Genes	Pathway	Enrichment	Genes
Transition between fast and slow fiber	271.381	TNNT1 TNNI1 ACTN3	Nucleosome positioning	168.8592593	H1-1, H1-5
Reg. of skeletal muscle adaptation	146.1282	TNNT1 TNNI1 ACTN3	Establishment of skin barrier	101.3155556	FLG, HRNR
Skeletal muscle adaptation	105.537	TNNT1 TRIM63 TNNI1 ACTN3	Reg. of water loss via skin	93.81069959	FLG, HRNR
Muscle filament sliding	61.77778	TNNT1 TNNI1 MYL4 ACTN3	Cornification	30.15343915	FLG, TCHH KRT73
Actin–myosin filament sliding	61.77778	TNNT1 TNNI1 MYL4 ACTN3	Keratinization	18.76213992	FLG TCHH KRT73 HRNR
Striated muscle adaptation	57.56566	TNNT1 TRIM63 TNNI1 ACTN3	Keratinocyte differentiation	14.47365079	
Reg. of muscle adaptation	30.51673	TNNT1 TRIM63 TNNI1 ACTN3	Epidermal cell differentiation	12.03272631	
Muscle adaptation	23.89518	TNNT1 TRIM63 TNNI1 ACTN3	Skin development	10.73258004	
Muscle contraction	10.52447	TNNT1 TRIM63 TNNI1 MYL4 ACTN3 FXYD1	Epidermis development	9.558071279	
Generation of precursor metabolites and energy	7.762794	MT3 TYRP1 ENO3 GYS2 SLC25A4 COX7A1 ACTN3			

**Table 3:** Comparison of cellular components of upregulated and downregulated genes between OSMF-derived OSCC and de novo OSCC groups.

Pathway	Enrichment of Upregulated Genes	Genes
Troponin complex	115.1313131	TNNT1 TNNI1
Melanosome membrane	70.35802469	DCT TYRP1
Chitosome	70.35802469	DCT TYRP1
Pigment granule membrane	70.35802469	DCT TYRP1
Sarcomere	17.19155354	TNNT1 TRIM63 TNNI1 ABRA MYL4 ACTN3
Myofibril	15.57103825	TNNT1 TRIM63 TNNI1 ABRA MYL4 ACTN3
Contractile fiber	15.0171278	TNNT1 TRIM63 TNNI1 ABRA MYL4 ACTN3
Actin cytoskeleton	7.009840098	TNNT1 GYS2 TNNI1 ABRA MYL4 ACTN3
Supramolecular polymer	4.563763764	MT3 TNNT1 TRIM63 TNNI1 ABRA KRT76 MYL4 ACTN3
Supramolecular complex	3.916838488	DDX3Y MT3 TNNT1 TRIM63 TNNI1 ABRA KRT76 MYL4 ACTN3
**Pathway**	**Enrichment of Downregulated Genes**	**Genes**
Keratohyaline granule	633.2222222	FLG HRNR
PET complex	422.1481481	TDRD12
Cornified envelope	75.98666667	FLG TCHH HRNR
Nucleosome	33.03768116	H1-1 H1-5 H2AC12
DNA packaging complex	30.88888889	H1-1 H1-5 H2AC12
Protein–DNA complex	17.26969697	H1-1 H1-5 H2AC12

**Table 4:** Clinical and pathological characteristics of all samples.

Sl. No.	Age	Gender	Anatomical Site	TNM Stage	Histopathologic Diagnosis Based on Tumor Grade
1	42	Female	Left Buccal Mucosa	T_1_N_0_M_0_	Well differentiated Oral Squamous Cell Carcinoma
2	63	Female	Left Buccal Mucosa	T_1_N_0_M_0_	Well differentiated Oral Squamous Cell Carcinoma
3	63	Female	Right Buccal Mucosa and Alveolus	T_1_N_0_M_0_	Well differentiated Oral Squamous Cell Carcinoma
4	60	Male	Right Lateral Border of Tongue	T_1_N_0_M_0_	Well differentiated Oral Squamous Cell Carcinoma
5	77	Female	Right Gingivobuccal Sulcus	T_2_N_1_M_0_	Moderately differentiated Oral Squamous Cell Carcinoma
6	65	Female	Left Buccal Mucosa and Alveolus	T_1_N_0_M_0_	Microinvasive Oral Squamous Cell Carcinoma
7	41	Male	Right Buccal Mucosa	T_1_N_0_M_0_	Well differentiated Oral Squamous Cell Carcinoma
8	64	Male	Left Gingivobuccal Sulcus	T_2_N_1_M_0_	Moderately differentiated Oral Squamous Cell Carcinoma
9	48	Female	Right Buccal Mucosa	T_1_N_0_M_0_	Oral Submucous Fibrosis-derived Oral Squamous Cell Carcinoma
10	30	Male	Right Buccal Mucosa	T_1_N_0_M_0_	Oral Submucous Fibrosis-derived Oral Squamous Cell Carcinoma
11	41	Male	Left Buccal Mucosa	T_1_N_0_M_0_	Oral Submucous Fibrosis-derived Oral Squamous Cell Carcinoma
12	48	Male	Left Buccal Mucosa	T_1_N_0_M_0_	Oral Submucous Fibrosis-derived Oral Squamous Cell Carcinoma
13	60	Female	Right Buccal Mucosa	T_1_N_0_M_0_	Oral Submucous Fibrosis-derived Oral Squamous Cell-Carcinoma

## Data Availability

The data that support the findings of this study are available within the manuscript and as supplementary data uploaded to the GEO repository with accession number GSE319719, viewed at https://www.ncbi.nlm.nih.gov/geo/query/acc.cgi?acc=GSE319719 (accessed on 17 February 2026). All shared data comply with ethical approvals and participant consent. It is currently private and is scheduled to be released on 2 June 2026.

## Abbreviations

The following abbreviations are used in this manuscript:

OSMF	Oral Submucous Fibrosis
OSCC	Oral Squamous Cell Carcinoma
NEXTflex	Next-Generation Flexible Hybrid Electronics
UCSC hg19	University of California, Santa Cruz human genome version 19
DEGs	Differentially Expressed Genes
GO	Gene Ontology
lncRNAs	Long non-coding RNAs
miR-31	Micro RNA-31
miR-21	Micro RNA-21
miR-29b	Micro RNA-29b
GSTM1	Glutathione S-Transferase Mu 1
GSTT1	Glutathione S-Transferase Theta 1
RNA	Ribo Nucleic Acids
RIN	RNA Integrity Number
RNA-	RNA-Seq Kit—RNA Sequencing Kit
cDNA	Complementary Deoxyribonucleic Acid
dUTP	Deoxyuridine Triphosphate
PCR	Polymerase Chain Reaction
GRCh37	Genome Reference Consortium Human Build 37
KEGG	Kyoto Encyclopedia of Genes and Genomes
PET complex	Photosynthetic Electron Transport
DNA	Deoxyribonucleic Acid
PET-CT	Positron Emission Tomography–Computed Tomography
Rho GTPases	Ras Homologous Guanosine Triphosphatases
ABPs	Actin-Binding Proteins
RhoA	Ras Homolog Family Member A
ADSCs	Adipose-Derived Stem Cells
CACNG7	Calcium Voltage-Gated Channel Auxiliary Subunit Gamma 7
MKRN9	Makorin Ring Finger Protein 9
RSPO	R-Spondin
DCT	Dopachrome Tautomerase
DHICA	5,6-Dihydroxyindole-2-Carboxylic Acid
